# Risk Factors for the Development of Nerve Palsy Following Primary Total Hip Arthroplasty

**DOI:** 10.2174/1874325001812010164

**Published:** 2018-04-23

**Authors:** Shunsuke Kawano, Motoki Sonohata, Masaru Kitajima, Masaaki Mawatari

**Affiliations:** Department of Orthopaedic Surgery, Faculty of Medicine, Saga University, 5-1-1 Nabeshima, Saga 849-8501, Japan

**Keywords:** Risk factor, Total hip arthroplasty, Nerve palsy, Sciatic nerve, Complication, Clinical result

## Abstract

**Background:**

Nerve palsy following total hip arthroplasty (THA) is a complication that worsens the functional prognosis. The present study analyzed the risk factors of nerve palsy following THA.

**Methods:**

The subjects of this study included 6,123 cases in which primary THA was performed under spinal anesthesia with cementless implants used in the posterolateral approach.

**Results:**

Fourteen cases (0.22%) developed nerve palsy following THA, all of which involved palsy of the entire peroneal nerve region. The diagnoses included osteoarthritis due to subluxation (n=6), complete hip dislocation (n=3), osteonecrosis of the femoral head (n=2), primary osteoarthritis (n=1), osteoarthritis due to trauma (n=1), and multiple osteochondromatosis (n=1). Recovery from nerve palsy was confirmed in 10 cases; the longest recovery period was three years. A univariate analysis revealed significant differences in the osteoarthritis due to subluxation, osteonecrosis of the femoral head, complete hip dislocation, body weight and body mass index. However, none of the factors remained significant in multivariate analysis. Peroneal (ischiadic) nerve palsy following THA occurred in patients with osteonecrosis of the femoral head, complete hip dislocation, low body weight and a low body mass index. However, there were no cases of nerve palsy after the introduction of THA combined with shortening osteotomy of the femur for complete hip dislocation. patients.

**Conclusion:**

It is necessary to pay attention to direct pressure in cases of lower body weight and lower BMI because compression of the sciatic nerve during surgery and compression of the fibular head are considered to be risk factors.

## 1 INTRODUCTION

The complications associated with Total Hip Arthroplasty (THA) include dislocation, infection, looseness, blood clotting, embolisms, fracturing, vascular injury, and nerve injury (nerve palsy). In the short term, dislocation and infection may cause revision THA and the rate of revision THA due to loosening increases during the long-term follow-up [1]. While nerve palsy after THA has little effect on the long-term fixation of implants, preventive measures should be implemented against this complication because it worsens the functional prognosis.

The incidence of nerve palsy following THA is reported to range from 0.08% to 7.6%; the frequency of occurrence and the nerves that are affected vary depending on the approach [2-9]. Nerve palsy occurs at sites that are under pressure from retractors during surgery, sites of direct injury during the insertion of the implant, and due to the nerve extension that occurs in association with the elongation of the leg length; however, there are also many cases in which the cause of occurrence is unclear and these represent catastrophic complications. The morphology of the nerve damage is associated with one or a combination of factors, which include compression, ischemia, extension, crushing, and tearing [3, 10]. Sciatic nerve palsy is the most common type of nerve damage, followed by femoral nerve palsy, and the combination of sciatic and femoral nerve palsy; paralysis of the obturator nerve is uncommon [11, 12]. In recent years, there have also been reports of lateral femoral cutaneous nerve palsy in association with the direct anterior approach [13].

Several reports have been published on the risk factors for nerve palsy following the excessive elongation of the leg length, trauma and previous surgery of hip, revision THA, osteoarthritis due to subluxation, cement indentation, pressure from retractors during operation, postoperative hematoma, usage of wires in the trochanter fixation, young age, female sex, longer operation time and the posterolateral approach [3, 5-8, 10, 12, 14-16]. One study on nerve palsy following THA reported that the odds ratio for the risk of nerve palsy in osteoarthritis due to subluxation is 3.7, while that for osteoarthritis due to trauma is 3.8 [12].

The aim of the present study was to analyze the incidence of and risk factors for nerve palsy in a consecutive series of Japanese patients who underwent cementless THA using the posterolateral approach.

The study protocol adhered to the ethical guidelines of the 1975 Declaration of Helsinki, and the institutional review board of our institution approved this study (2017-04-26).

## 2 METHODS

The subjects of the study consisted of 6,123 cases from among 4,095 patients who underwent primary THA at this hospital from September 1998 to December 2013. These included 959 cases in 810 male patients and 5,164 cases in 4,085 female patients. The average age at the time of surgery was 62.1 years (range, 18–98 years). THA was performed using the posterolateral approach under spinal anesthesia with identical cementless implants (AMS-HA acetabular shell and PerFix-HA stem; Kyocera, Osaka, Japan). Three cases in which primary THA was performed using cement implants for osteopetrosis and cases of revision THA were excluded from the present study.

The number of cases of postoperative nerve palsy, the diagnosis, the amount of leg elongation and the prognosis were investigated. Two groups were created, one in which postoperative nerve palsy occurred and one in which it did not, and the risk factors for nerve palsy were analyzed. A univariate analysis was conducted to analyze the risk factors for nerve palsy, with the dependent variable being whether or not palsy occurred and the independent variables being age, gender, height, weight, body mass index (BMI), affected side, diagnosis, range of motion (ROM) before the operation, and the Japanese Orthopaedic Association (JOA) Hip Score before the operation, the operative time, operative bleeding, and leg elongation, which are considered to risk factors for the nerve palsy. The JOA hip score is used to evaluate the hip joint function. It has four categories, with a maximum total score of 100 points: pain (40 points), range of motion (20 points), walking ability (20 points), and activities of daily living (20 points). The amount of leg elongation was determined as the difference in the distance from the superior anterior iliac spine to the medial malleolus of the ankle before and after the operation. Variables that showed statistical significance in the univariate analysis were included in a multivariate analysis (regression analysis). Furthermore, nerve palsy was diagnosed with less than a Manual Muscle Test (MMT) grade 2 and sensory disorder of the proprioceptive sensation regions. In this study, complete nerve palsy was diagnosed based on an MMT grade of 0 and incomplete nerve palsy was diagnosed based on an MMT grade of 1 or 2. Furthermore, the cases involving complete hip dislocation (Crowe IV) were analyzed separately from those involving osteoarthritis due to subluxation. Eight early cases of complete hip dislocation underwent THA without femoral shortening osteotomy at the judgment of the operator, while the other cases of complete hip dislocation underwent THA with femoral shortening osteotomy.

The IBM SPSS Statistics software program (version 19 for Windows; SPSS Inc., an IBM Company, Chicago, IL, USA) was used to perform the statistical analyses. Student’s *t*-test, the chi-square test, and a logistic regression analysis were carried out. P values of < 0.05 were considered to indicate statistical significance.

## 3 RESULTS

Nerve palsy occurred following primary THA in 14 out of 6,123 (0.22%) cases, all of which involved nerve palsy of the entire peroneal nerve region. The diagnoses included osteoarthritis due to subluxation (n=6), complete hip dislocation (n=3), osteonecrosis of the femoral head (n=2), primary osteoarthritis (n=1), osteoarthritis due to trauma (n=1), and multiple osteochondromatosis (n=1). The extent of nerve palsy included incomplete paralysis (n=8) and complete paralysis (n=6). The mean leg elongation of all cases was 1.33 cm (range, 0 to 6.5 cm), and the value in palsy cases was 1.96 cm (range, 0.5 to 4.5 cm) while that in no palsy cases was 1.96 cm (range, 0.5 to 4.5 cm). The mean leg elongation of complete hip dislocation cases without femoral shortening osteotomy was 4.8 cm (range, 3.0 to 6.5 cm), and the mean leg elongation of complete hip dislocation with femoral shortening osteotomy was 3.1 cm (range, 2.0-4.0 cm). Additional operations included leg shortening by stem revision (n=1) and neurolysis (n=1). The muscular strength recovered to more than an MMT score of 3 in the 8 cases of incomplete paralysis. One of the 6 cases involving complete paralysis recovered to more than an MMT score of 3 and 1 recovered to an MMT score of 2; the remaining 4 cases showed no recovery, even after three years. In the cases in which the patients recovered, a period of 1 to 36 months was required for the muscle strength to recover. In cases in which stem revision was performed as the additional operation, the diagnosis was complete hip dislocation and a leg length extension (45 mm) was performed. Incomplete paralysis occurred; however, the neurological disorder recovered by shortening the legs during stem revision. In cases in which neurolysis was performed, the pain and numbness at the distal end of the fibulae head were strong, and neurolysis of the fibulae head was performed. While the pain and numbness subsided, the motor palsy showed no improvement in the two years after the operation (Table **1**).

In the analysis of the risk factors for nerve palsy, there were no significant differences in age, gender, affected side, ROM, JOA hip score, operation time, the bleeding of operation, and the leg elongation; however, significant differences were confirmed in the diagnosis (osteoarthritis due to subluxation, osteonecrosis of the femoral head, complete hip dislocation), weight and BMI (Table **2**). None of the risk factors remained significant in the multivariate analysis (Table **3**). In the cases involving osteonecrosis of the femoral head, the ROM before operation was good and the leg length discrepancy was small; however, there were many cases involving patients with a relatively low body weight and relatively low BMI (Table **4**).

## 4 DISCUSSION

This study is a report on 6123 cases from the same institution. In all cases, THA was performed using the posterolateral approach with the same cementless implant. Furthermore, all of the subjects were Japanese and an analysis of the risk factors for nerve palsy was conducted.

In the present study, the incidence of nerve palsy following primary THA was 0.22%, with all cases of nerve palsy involving the entire peroneal nerve region. Only three cases showed factors that were considered to be associated with the occurrence of nerve palsy. The diagnosis was complete hip dislocation in each case and excessive elongation of the leg length was performed. Femoral shortening osteotomy was not performed in these cases and leg length elongation of more than 30 mm was performed during the operation. Thus, it is suspected that nerve palsy occurred due to excessive leg elongation.

Recovery from nerve palsy was observed in 10 of the 14 cases (71%) in the present study, and no recovery was observed in the four cases of complete paralysis. The average time until recovery was 10.8 months (range, 1 to 36 months). The recovery rate from nerve palsy following THA differs according to the degree of paralysis. Approximately 90% of incomplete paralysis recover. In contrast only 40–50% of complete paralysis show a complete recovery. In many cases, the period of recovery was within two years, and an incomplete nerve palsy was associated with an earlier. Sciatic nerve palsy tends to require a longer time for recovery than the other nerves, and there is one reported case in which recovery was judged to have occurred from 5 to 7 years after a THA [17-19]. According to Jacob et. al., nerve palsy following THA was confirmed in 93 of 12,998 cases (0.72%), with a complete recovery occurring in 46 cases (50%). The period until recovery was less than 6 months in 17 cases (37%), 6 to12 months in 17 cases (37%), and more than 1 year in 12 cases (24%). Furthermore, 4 cases (4%) did not recover until a mean of 2.2 years after the operation [15].

Nerve palsy after THA occurred significantly more frequently in patients with a lower body weight and a lower BMI in the present study. The sciatic nerve was present near the operative field with the posterolateral approach, and direct pressure was applied during the operation. Thus, sciatic nerve palsy may have occurred due to crushing or ischemia. In cases with a lower body weight and lower BMI, it is easy for direct pressure to be applied to the sciatic nerve, and there were probably significant differences in the incidence of nerve palsy. Furthermore, because operations were performed under spinal anesthesia in the present study, peroneal nerve palsy due to the direct pressure of the fibular head during the postoperative resting period may have developed easily in patients with a lower body weight and lower BMI. It is possible that anesthesia may cause nervous injury when using spinal anesthesia or nerve block. However, one study reported that there was no difference in the incidence of nerve disorders resulting from anesthetization in THA [15].

The risk of nerve palsy is reported to be high in patients with osteoarthritis due to subluxation because the sciatic nerve is close to the ischium and ilium [12, 20]. However, the incidence of paralysis was significantly low in patients with osteoarthritis due to subluxation in the present study. This was influenced by the fact that all of the subjects were Japanese and that osteoarthritis due to subluxation was the most common diagnosis [21, 22]. Furthermore, the fact that that complete hip dislocation was not confused with osteoarthritis due to subluxation likely influenced this result. In contrast, nerve palsy occurred in three cases of complete hip dislocation and was significantly more common in cases of complete hip dislocation than other diagnosis. The three cases of complete hip dislocation that developed nerve palsy did not undergo femoral shortening osteotomy, and the longest leg elongation was 45 mm. Similarly to Sonohata's report, there were no cases of nerve palsy in patients with complete hip dislocation who underwent THA with femoral shortening osteotomy [23, 24].

The incidence of osteonecrosis of the femoral head was significantly high. The body weight and BMI were significantly lower in the osteonecrosis cases than in other diagnosis cases (Table **4**). Because a lower body weight and lower BMI were found to be risk factors of nerve palsy after THA in this study, we believe that osteonecrosis of the femoral head also represents a risk factor of nerve palsy after THA.

Many studies have reported that the excessive elongation of the leg length is a risk factor for nerve palsy following THA [5, 7, 8, 16]. However, determining the leg elongation length at which paralysis will not occur is difficult [19]. The length of leg elongation in the cases that developed nerve palsy did not exceed 20 mm except for in cases of complete hip dislocation and osteoarthritis for trauma. In the present study, nerve palsy even occurred in cases in which the length of leg elongation was small and leg elongation was not a significant factor. We consider that the nerve palsy after THA occurs by a multiple factorial.

The present clinical study was associated with some limitations. The study was a retrospective study and nerve palsy at the sciatic nerve part or nerve palsy by the fibula head could not be ruled out in cases of peroneal nerve palsy. The differentiation between palsy of the sciatic nerve and the peroneal nerve can be made by electromyography of the short head of the biceps femoris. However, this was not implemented in the present study. Another limitation is the possibility that minor nerve palsy was overlooked.　Furthermore, the procedure used to measure the leg length is a simple method limited only by its inability to reflect the true bony length.

## CONCLUSION

Nerve palsy occurred in 14 out of 6,123 Japanese cases (0.22%) in which primary THA was performed using the post lateral approach. In all cases the entire peroneal nerve region was affected. Recovery from nerve palsy was confirmed in 10 of the 14 cases (71.4%) with recovery requiring a maximum of 36 months. The univariate analysis revealed that complete hip dislocation, osteonecrosis of the femoral head, low body weight, and low BMI were risk factors for nerve palsy following THA. It is important to make efforts to prevent paralysis because the recovery from nerve palsy and the recovery of the leg function is delayed when nerve palsy occurs. In THA using posterolateral approach, it is necessary to pay attention to the pressure on the sciatic nerve during surgery and pressure on the fibular head before the recovery from postoperative spinal anesthesia when the operation is performed using spinal anesthesia. Furthermore, more care should be taken in cases involving patients with a low body weight and a low BMI.

## Figures and Tables

**Table 1:** Cases of nerve palsy in peroneal nerve reision.

	**Age** **(year)**	**Sex**	**Diagnosis**	**Leg Elongation** **(mm)**	**Palsy Degree**	**Prognosis**		
						**Recovery**	**Period**	**Additional Surgery**
**1**	58	F	Completely Dislocated Hip	45	incomplete	full	1 m	
**2**	56	F	Osteoarthritis for Trauma	35	incomplete	full	2 m	
**3**	51	F	Completely Dislocated Hip	40	incomplete	full	2 y	Stem Revision
**4**	55	F	Osteoarthritis for Subluxation	15	incomplete	full	2 m	
**5**	42	F	Osteonecrosis of the Femoral Head	20	incomplete	full	6 m	
**6**	74	F	Completely Dislocated Hip	35	incomplete	full	1 y	
**7**	64	F	Osteochondromatosis	5	complete	no	- (2 y)	Neurolysis
**8**	68	F	Osteoarthritis for Subluxation	10	complete	imperfection	- (7 y)	-
**9**	52	F	Osteoarthritis for Subluxation	15	complete	full	3 y	-
**10**	60	F	Osteoarthritis for Subluxation	20	complete	no	- (4 y)	-
**11**	64	F	Primary Osteoarthritis	10	complete	no	- (3 y)	-
**12**	62	F	Osteonecrosis of the femoral Head	10	complete	no	- (2 y)	-
**13**	53	F	Osteoarthritis for Subluxation	20	incomplete	full	3 y	-
**14**	60	F	Osteoarthritis for Subluxation	10	incomplete	full	3 m	-

F: female m: month y: year

**Table 2:** Subject Characteristics and results of analysis for the factors influencing nerve palsy.

	**All Patients** **(N = 6123)**	**Palsy Patients** **(N = 14)**	**Non-Palsy Patients** **(N = 6109)**	**P Value**
**Age (year)** **(mean ± SD)**	62.0±10.9	58.5± 8.0	62.1± 10.9	0.11
** Gender (Male: Female)**	959:5164	0:14	959:5150	0.11
**Body Height (cm)** **(mean ± SD)**	153.6±46.0	151.5± 6.2	153.6± 46.2	0.24
**Body Weight (Kg)** **(mean ± SD)**	55.7±10.3	52.0± 7.8	55.8± 10.4	0.04
**Body Mass Index (Kg/m^2^)** **(mean ± SD)**	24.7±11.9	22.7± 3.0	24.7± 12.0	0.04
** Affected side (left: right)**	2980:3143	8:6	2972:3137	0.53
** Pre-operative Range of Motion (degree)**				
** Flexion (mean ± SD)**	77.5±25.5	75.7± 29.9	77.6± 25.5	0.82
** Extension (mean ± SD)**	-5.3±10.1	-7.9± 11.6	-5.2± 10.1	0.40
** Abduction (mean ± SD)**	17.3±11.6	16.4± 9.1	17.3± 11.6	0.72
** Adduction (mean ± SD)**	12.6±8.4	12.9± 11.9	12.6± 8.3	0.94
** External Rotation** **(mean ± SD)**	24.3±15.9	22.5± 21.6	24.3± 15.8	0.77
** Internal Rotation** **(mean ± SD)**	7.1±20.8	15.0± 24.2	7.0± 20.7	0.24
****Diagnosis (number of joint)****				
** Osteoarthritis for subluxation**	4752	7	4745	0.01
** Primary Osteoarthritis**	234	1	233	0.52
** Completely Dislocated Hip**	359	3	356	0.01
** Osteoarthritis for Perthes disease**	18	0	18	0.84
** Osteoarthritis for trauma**	129	1	128	0.19
** Rheumatoid Arthritis**	93	0	93	0.64
** Osteonecrosis of the femoral head**	235	2	233	0.04
** Rapidly Destructive Coxarthropathy**	175	0	175	0.81
** Ankylosed Hip Joint**	107	0	107	0.61
** Skeletal Dysplasia**	14	0	14	0.86
** Previous piogenic arthritis**	38	0	38	0.77
** Previous hip Osteotomy**	528	1	527	0.84
** Pre-operative JOA Hip score**				
** Total (mean ± SD)**	47.0± 13.8	49.1± 8.8	47.0± 13.8	0.42
**Pain (mean ± SD)**	15.7± 8.5	16.9± 8.5	15.7± 8.5	0.62
**Gait (mean ± SD)**	9.2± 3.9	9.6± 3.8	9.2± 3.9	0.69
**ROM (mean ± SD)**	10.9± 4.8	10.4± 4.8	10.9± 4.8	0.67
**ADL (mean ± SD)**	24.3±15.9	12.2± 3.9	11.4± 3.1	0.51
** Surgical Factors**				
** Operation time (min)** **(mean ± SD)**	44.4± 19.2	50.2± 16.0	44.3± 19.2	0.19
** Operation bleeding (g)** **(mean ± SD)**	240.3± 153.0	267.7± 216.5	240.2± 152.9	0.64
** Post-Operation bleeding (g)** **(mean ± SD)**	467.8± 275.2	524.8± 323.7	467.7± 275.1	0.52
** Total bleeding (g)** **(mean ± SD)**	682.4± 329.4	792.5± 417.9	682.3± 329.3	0.34
**leg delongation (cm)** **(mean ± SD)**	1.34± 1.37	1.96± 1.22	1.33± 1.37	0.08

SD: Standard Deviation.

JOA: Japan Orthopaedic Association

ROM: Range of Motion

ADL: Activities of Daily Living

**Table 3:** Odds of the risk factors influencing nerve palsy as indicated by multivariable regression analysis.

**Variable**	**Odds Ratio**	**95% Confidence Interval**		**P value**
		Lower	Upper	
**Body Weight (Kg)** **(mean ± SD)**	0.98	0.89	1.07	0.59
**Body Mass Index (Kg/m^2^)** **(mean ± SD)**	0.96	0.77	1.21	0.75
**Osteoarthritis for subluxation**	0.60	0.12	2.91	0.53
**Completely Dislocated Hip**	3.04	0.49	18.64	0.23
**Osteonecrosis of the femoral head**	3.51	0.46	26.44	0.22

SD: Standard Deviation.

**Table 4:** Subject characteristics of analysis for the ION patients.

	**All Patients** **(N = 6123)**	**ION Patients** **(N = 235)**	**Non-ION Patients** **(N = 5888)**	**P Value**
**Age (year)** **(mean ± SD)**	62.0±10.9	55.3± 14.7	62.4± 10.7	<0.01
**Gender (Male: Female)**	959:5164	131:104	828:5060	<0.01
**Body Height (cm)** **(mean ± SD)**	153.6±46.0	165.6± 9.3	153.2± 43.4	0.04
**Body Weight (Kg)** **(mean ± SD)**	55.7±10.3	59.0± 12.2	55.6± 10.3	<0.01
**Body Mass Index (Kg/m^2^)** **(mean ± SD)**	24.7±11.9	22.9± 4.1	24.3± 12.2	<0.01
**Leg Length Discrepancy (mm)** **(mean ± SD)**		-0.3± 4.9	-0.8± 13.9	0.12
**Affected side (left: right)**	2980:3143	124:111	2856:3032	0.21
**Pre-operative Range of Motion (degree)**				
**Flexion (mean ± SD)**	77.5±25.5	87.5± 25.8	77.2± 25.4	<0.01
**Extension (mean ± SD)**	-5.3±10.1	-2.1± 8.0	-5.3± 10.1	<0.01
**Abduction (mean ± SD)**	17.3±11.6	18.3± 11.7	17.3± 11.6	0.18
**Adduction (mean ± SD)**	12.6±8.4	14.0± 8.8	12.6± 8.3	0.01
**External Rotation** **(mean ± SD)**	24.3±15.9	25.2± 16.7	19.8± 15.8	<0.01
**Internal Rotation** **(mean ± SD)**	7.1±20.8	5.9± 10.9	7.1± 15.9	0.09
**Pre-operative JOA Hip score**				
**Total (mean ± SD)**	47.0± 13.8	47.7± 15.4	47.0± 13.7	0.49
**Pain (mean ± SD)**	15.7± 8.5	14.9± 8.2	15.7± 8.5	0.16
**Gait (mean ± SD)**	9.2± 3.9	9.3± 4.5	9.2± 3.8	0.65
**ROM (mean ± SD)**	10.9± 4.8	12.2± 4.6	10.9± 4.8	<0.01
**ADL (mean ± SD)**	24.3±15.9	11.4± 3.8	11.4± 3.1	0.92
**Surgical Factors**				
**Operation time (min)** **(mean ± SD)**	44.4± 19.2	41.6± 14.9	44.4± 19.4	0.03
**Operation bleeding (g)** **(mean ± SD)**	240.3± 153.0	215.6± 147.4	241.2± 153.3	0.01
**Post-Operation bleeding (g)** **(mean ± SD)**	467.8± 275.2	511.6± 295.6	466.1± 274.2	0.02
**Total bleeding (g)** **(mean ± SD)**	682.4± 329.4	694.5± 352.1	682.1± 328.6	0.59

SD: Standard Deviation. JOA: Japan Orthopaedic Association ROM: Range of Motion ADL: Activities of Daily Living

## LIST OF ABBREVIATIONS

BMI: = Body Mass Index
JOA: = Japanese Orthopaedic Association
ROM: = Range of Motion
THA: = Total Hip Arthroplasty
